# Diagnostic utility of ultra-microangiography and shear wave elastography in pediatric carpal tunnel syndrome associated with mucopolysaccharidosis

**DOI:** 10.1007/s00247-026-06567-5

**Published:** 2026-03-07

**Authors:** Nusret Seher, Banu Kadıoğlu Yılmaz, Ayça Burcu Kahraman, İsmail Dilek, Haluk Gümüs, Mehmet Öztürk

**Affiliations:** 1 Department of Radiology, Selçuk Üniversitesi Tıp Fakültesi Hastanesi, Konya, Turkey; 2 Division of Pediatric Metabolism, Department of Pediatrics, Selçuk Üniversitesi Tıp Fakültesi Hastanesi, Konya, Turkey; 3 Division of Pediatric Metabolism, Konya City Hospital, Konya, Turkey; 4 Department of Neurology, Selçuk Üniversitesi Tıp Fakültesi Hastanesi, Konya, Turkey

**Keywords:** Mucopolysaccharidosis, Carpal tunnel syndrome, Ultra-microangiography, Shear wave elastography, Median nerve, Pediatric ultrasonography

## Abstract

**Background:**

Carpal tunnel syndrome is rare in the paediatric population but is frequently associated with mucopolysaccharidosis. Owing to nonspecific symptoms and limited reliability of clinical examination, early diagnosis of carpal tunnel syndrome in patients with mucopolysaccharidosis remains challenging. Advanced ultrasonographic techniques may enable objective and early detection.

**Objective:**

To evaluate the diagnostic performance of ultra-microangiography and shear wave elastography for the early detection of carpal tunnel syndrome in pediatric patients with mucopolysaccharidosis and to investigate their potential contribution to existing diagnostic protocols.

**Materials and methods:**

This cross-sectional study included 44 individuals (mean age, 11.6±4.8 years), comprising 22 pediatric patients with genetically confirmed mucopolysaccharidosis and 22 age- and sex-matched healthy controls. The mucopolysaccharidosis group was further divided into carpal tunnel syndrome–negative (*n*=16) and carpal tunnel syndrome–negative (*n*=6) subgroups based on electromyography. Median nerve cross-sectional area, vascular index obtained by ultra-microangiography, and stiffness values measured by shear wave elastography (expressed in kilopascals) were evaluated bilaterally. Diagnostic performance was assessed using receiver operating characteristic analysis.

**Results:**

Significant differences were observed among control, carpal tunnel syndrome-negative mucopolysaccharidosis, and carpal tunnel syndrome-positive mucopolysaccharidosis groups for median nerve cross-sectional area, vascular index, and stiffness values (all *P*<0.001). These parameters demonstrated high diagnostic performance in distinguishing mucopolysaccharidosis patients from healthy controls, with area under the curve values ranging from 0.853 to 1.000. Among mucopolysaccharidosis patients, these parameters also demonstrated high accuracy in differentiating carpal tunnel syndrome-positive from carpal tunnel syndrome-negative individuals (AUC, 0.891–0.984), with sensitivity ranging from 83.3% to 100% and specificity from 87.5% to 93.7%.

**Conclusion:**

Our findings suggest that ultra-microangiography and shear wave elastography may provide complementary quantitative information for the evaluation of early median nerve changes in pediatric patients with mucopolysaccharidosis, although further studies are required to validate the clinical utility of shear wave elastography in peripheral nerves.

**Graphical abstract:**

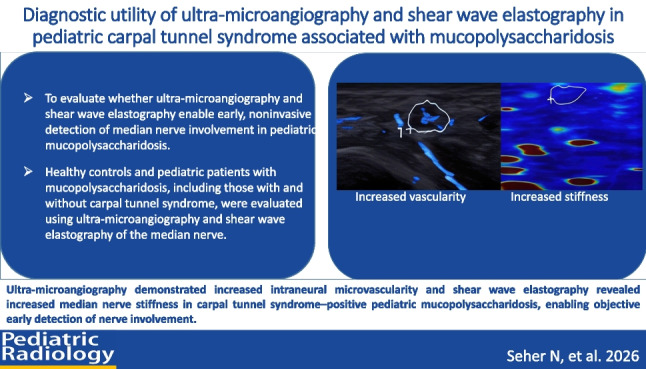

## Introduction

Mucopolysaccharidoses constitute a group of rare, genetically inherited metabolic disorders caused by a functional deficiency or marked insufficiency of one of the lysosomal hydrolase enzymes responsible for the degradation of glycosaminoglycans [1]. Owing to the wide spectrum of clinical manifestations and the partial overlap of phenotypes, seven distinct subtypes of mucopolysaccharidosis have been defined [2]. The disease leads to multisystem involvement, affecting the central and peripheral nervous systems, cardiopulmonary structures, the liver, the spleen, ocular tissues, and the musculoskeletal system [3].

Treatment options such as enzyme replacement therapy and hematopoietic stem cell transplantation have been shown to significantly improve cardiovascular function, cognitive capacity, and overall survival, particularly in selected patients [4]. However, the effects of enzyme replacement therapy on cardiac valve structures, the bronchial system, intraocular tissues, the vestibular system, and the central and peripheral nervous systems remain minimal.

This limitation leads to a more pronounced age-related progression of involvement in these systems over time [5]. Although evidence regarding the effectiveness of systemic therapies on musculoskeletal complications is limited, monitoring these findings with reliable screening methods is recommended based on studies demonstrating the benefits of early intervention [2, 6].

Carpal tunnel syndrome is a neuropathic condition caused by compression of the median nerve at the level of the carpal tunnel, where it courses together with the flexor tendons, and it represents one of the most common musculoskeletal complications within the mucopolysaccharidosis spectrum [7–9]. While carpal tunnel syndrome is the most common compression neuropathy in adults, it is encountered quite rarely in the pediatric population [10–12]. In pediatric cases, the most common underlying causes are lysosomal storage diseases, with mucopolysaccharidosis being particularly prominent [13]. In patients with mucopolysaccharidosis, the early signs and symptoms of carpal tunnel syndrome are often nonspecific and do not reflect the classic clinical presentation observed in adults [2]. As a result, the diagnosis is often established only after the development of thenar atrophy or significant impairment of hand function [14]. When these nonspecific early symptoms are combined with communication limitations related to age or intellectual disability, the diagnostic process may be delayed, ultimately resulting in permanent loss of hand function [15–17].

Therefore, various screening approaches have been proposed for the detection of carpal tunnel syndrome in patients with mucopolysaccharidosis; however, these methods exhibit significant variability in terms of sensitivity, specificity, and practical applicability [12, 18, 19]. In the current literature, there is no consensus regarding the optimal timing, frequency, or scope of carpal tunnel syndrome screening in patients with mucopolysaccharidosis. Determination of the severity of carpal tunnel syndrome is of critical importance for treatment planning. Previous studies have reported that patients with moderate carpal tunnel syndrome achieve more favorable outcomes following surgical intervention [20]. Therefore, early diagnosis plays a crucial role in achieving favorable treatment outcomes. Recent advances in ultrasonographic imaging have enabled improved assessment of tissue microvasculature without the use of contrast agents. Several imaging biomarkers have been developed for this purpose, including ultra-microangiography, superb microvascular imaging, and high-definition microvascular imaging. These techniques are based on advanced filtering algorithms that suppress tissue motion artifacts while preserving low-velocity blood flow signals, allowing more sensitive visualization of microvascular perfusion compared with conventional color or power Doppler imaging. Such approaches have gained increasing interest for the evaluation of inflammatory and fibrotic processes in musculoskeletal and peripheral nerve disorders.

Ultrasonography is a frequently preferred modality for nerve imaging due to its ease of use and noninvasive nature. In recent years, high-resolution ultrasonography techniques, which are more cost-effective and can be performed in a shorter time, have gained increasing prominence in the evaluation of compression neuropathies such as carpal tunnel syndrome [18, 21, 22]. Several advanced Doppler-based techniques, such as superb microvascular imaging and ultra-microangiography, have been developed to improve the visualization of low-velocity microvascular flow. These techniques are based on similar physical principles but are implemented using vendor-specific algorithms, and direct comparative studies between them in the evaluation of carpal tunnel syndrome are currently lacking.

Ultra-microangiography is a next-generation imaging technique that enhances the visualization of vascular structures without the need for contrast agent administration. Unlike conventional Doppler techniques, it employs advanced filtering algorithms capable of distinguishing low-velocity tissue motion from true blood flow, thereby increasing sensitivity to low-velocity hemodynamic signals [23]. UMA allows for both qualitative and quantitative assessments, and the vascular index (VI) is calculated based on the proportion of color pixels within the region of interest.

Shear wave elastography is an advanced ultrasonographic technique that enables noninvasive assessment of the mechanical properties of tissues, particularly tissue stiffness. This technique is based on the principle that the propagation velocity of shear waves increases with increasing tissue stiffness, enabling objective measurement of tissue elasticity expressed in kilopascals [24, 25].

The aim of this study was to evaluate the diagnostic performance and discriminatory power of next-generation ultrasonographic imaging techniques, including ultra-microangiography and shear wave elastography, for the early detection of carpal tunnel syndrome—which is frequently diagnosed at a late stage—in paediatric patients with a diagnosis of mucopolysaccharidosis, and to investigate the potential contribution of these methods to existing diagnostic protocols.

## Materials and methods

### Study design and ethical approval

This cross-sectional, observational study was conducted between February 2025 and July 2025 following approval from the institutional ethics committee of our university (Approval No: 2024/679). All procedures were performed in accordance with the principles of the Declaration of Helsinki. Written informed consent was obtained from the parents or legal guardians of all participants.

### Participants

The study group consisted of pediatric patients with a genetically confirmed diagnosis of mucopolysaccharidosis and age- and sex-matched healthy volunteers. Inclusion criteria comprised a confirmed diagnosis of any mucopolysaccharidosis subtype, an age range of 3–18 years, and clinical stability allowing for ultrasonographic evaluation. In accordance with the journal’s definition of pediatric age, all included participants were younger than 18 years at the time of examination. No patients or controls aged 18 years or older were included in the study cohort.

Exclusion criteria were as follows:

- Absence of a genetically confirmed diagnosis of mucopolysaccharidosis
- Presence of concomitant systemic diseases that could predispose to carpal tunnel syndrome, such as diabetes mellitus or hypothyroidism
- Lack of available electromyography examinations

All individuals in the mucopolysaccharidosis group underwent bilateral median nerve evaluation with electromyography performed by an experienced neurologist. The diagnosis of carpal tunnel syndrome was established based on electromyography findings, which served as the reference standard for group classification. Given the very low prevalence of carpal tunnel syndrome in the healthy paediatric population, only physical examination was performed in the control group, and electromyography was not conducted.

### Ultrasonography protocol

All imaging procedures were performed using a 9–14-MHz broadband linear array transducer (Mindray Bio-Medical Electronics, Shenzhen, China) by two radiologists (N.S. and M.O.) with 18 years and 10 years of experience in musculoskeletal ultrasonography, respectively. The imaging techniques included conventional B-mode ultrasonography, ultra-microangiography, and shear wave elastography. No sedation was administered.

Patients were positioned in the supine position to ensure optimal visualization of the median nerve at the level of the carpal tunnel inlet.

### Conventional ultrasonography

At the level of the carpal tunnel inlet, the cross-sectional area of the median nerve was measured by tracing the nerve within the epineurial borders. Three measurements were obtained from each hand, and the mean value was calculated for analysis. The total ultrasonographic examination time, including conventional ultrasonography, ultra-microangiography, and shear wave elastography, was approximately 10–15 min per patient.

### Ultra-microangiography

**Fig. 1:**
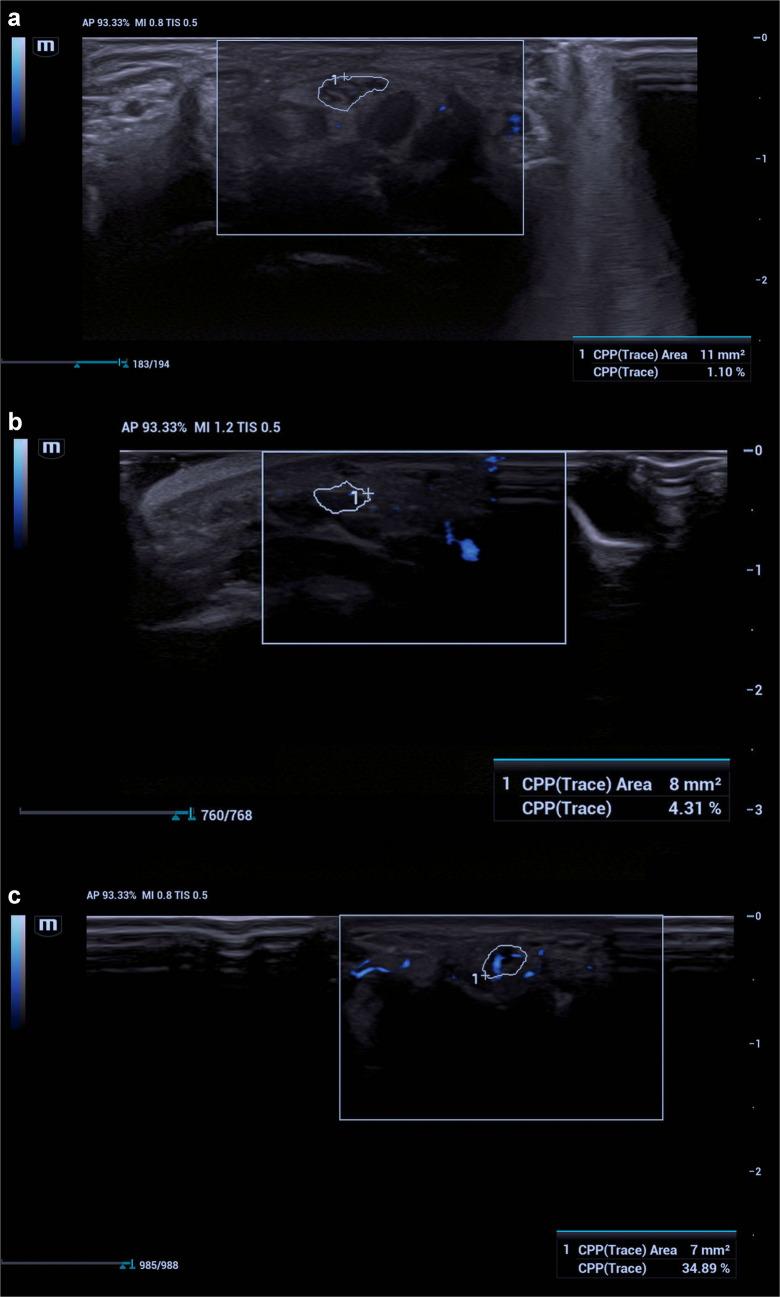
Comparative vascular index measurements obtained using ultra-microangiography at the level of the carpal tunnel. **a** Axial ultra-microangiography image of the median nerve in a healthy control (11-year-old boy), demonstrating minimal intraneural microvascular signal within the region of interest. **b** Axial ultra-microangiography image of the median nerve in a patient with mucopolysaccharidosis without carpal tunnel syndrome (10-year-old girl), showing mildly increased intraneural vascularity. **c** Axial ultra-microangiography image of the median nerve in a patient with mucopolysaccharidosis and electromyography-confirmed carpal tunnel syndrome (14-year-old boy), demonstrating increased intraneural microvascular signal

Ultra-microangiography was used to assess the microvascular architecture of the median nerve. This technique operates using highly sensitive Doppler algorithms and advanced filtering technologies that enable the detection of low-velocity blood flow [26]. Thus, even low-velocity blood flow that is difficult to detect with conventional color Doppler imaging can be clearly visualized. During the assessment, the transducer was positioned perpendicular to the skin surface in the axial plane, where the median nerve was best visualized, and probe pressure was kept to a minimum. A region of interest was manually drawn to encompass the entire nerve tissue. The ultra-microangiography software automatically calculated the density of color pixels (representing blood flow) detected within the region of interest. The vascular index was obtained as the percentage of color pixels relative to the total number of pixels within the region of interest (Fig. 1). This measurement semiquantitatively reflects perfusion at the level of the nerve microcirculation. During the examination, participants were instructed to keep their wrists in a neutral position, and the transducer was placed at the same anatomical level for each repetition. To improve measurement reproducibility, three consecutive measurements were obtained from both hands of each participant. To minimize potential variability among the three values, the mean vascular index value was used for statistical analysis. All ultra-microangiography measurements were performed by two independent radiologists experienced in musculoskeletal ultrasonography, and the final values were recorded based on a consensus agreement. Particular care was taken to apply minimal probe pressure during measurements to avoid vascular compression. All ultra-microangiography measurements were performed at the level of the carpal tunnel inlet, which represents the most commonly affected and anatomically standardized site for median nerve compression. Measurements were intentionally limited to a single standardized anatomical level to improve reproducibility and comparability between participants, as multi-level assessment may increase measurement variability, particularly in pediatric patients. Ultrasonographic image acquisition and measurements were performed independently by the two radiologists, who were not present in the examination room simultaneously and were blinded to each other’s measurements. Following independent assessments, the measurements were jointly reviewed, and final values were determined by consensus.

### Shear wave elastography

**Fig. 2:**
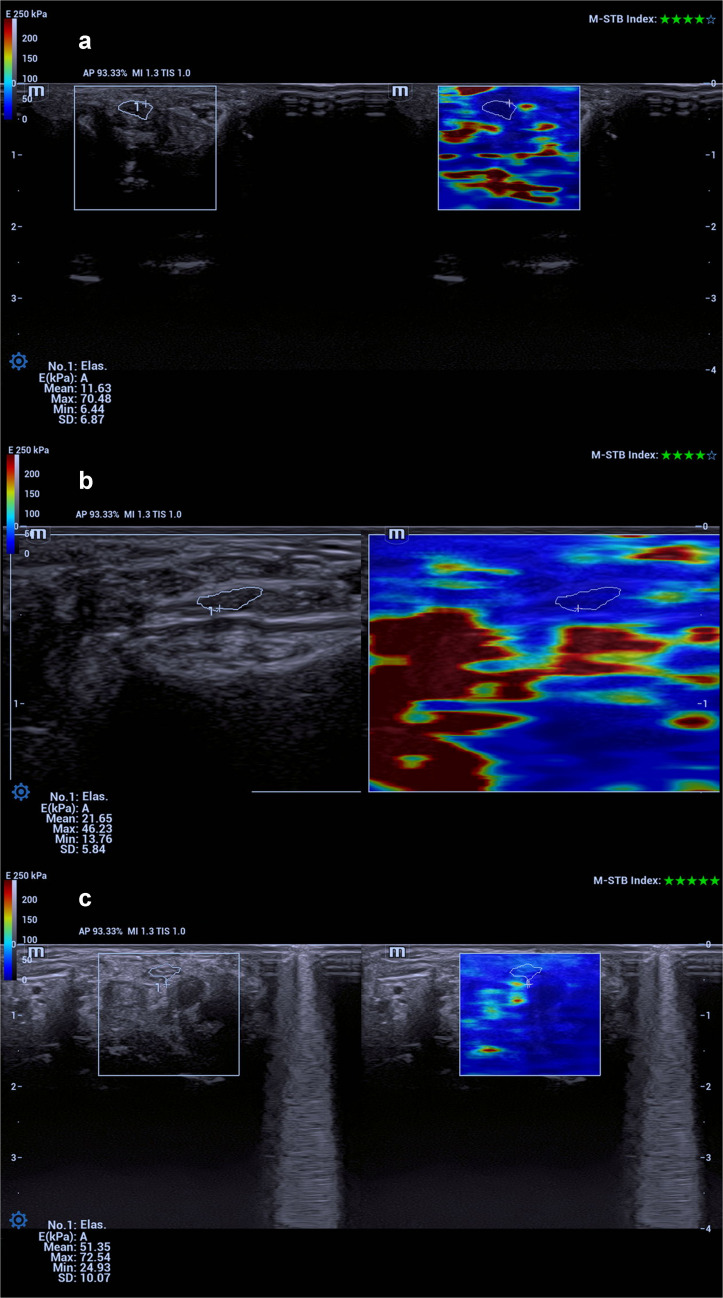
Shear wave elastography images of the median nerve obtained at the level of the carpal tunnel. **a** Longitudinal shear wave elastography image in a healthy control (12-year-old girl) demonstrating low stiffness values within the region of interest. **b** Longitudinal shear wave elastography image in a patient with mucopolysaccharidosis without carpal tunnel syndrome (9-year-old boy) showing moderately increased stiffness values. **c** Longitudinal shear wave elastography image in a patient with mucopolysaccharidosis and electromyography-confirmed carpal tunnel syndrome (15-year-old boy) demonstrating increased stiffness values expressed in kilopascals

Shear wave elastography is a quantitative elastography technique that enables the assessment of tissue mechanical properties based on the propagation velocity of shear waves generated by acoustic radiation force within the tissue [27]. In this technique, the measured shear wave propagation velocity is directly proportional to tissue stiffness, and the results are objectively expressed in kilopascals. During shear wave elastography measurements, the transducer was carefully positioned parallel to the long axis of the median nerve, and special care was taken to apply minimal pressure to the tissue throughout the examination. To avoid compression artifacts and falsely increased stiffness values, only gel contact between the probe and the skin was ensured during all measurements. After the median nerve was visualized in the longitudinal plane at the level of the carpal tunnel, a region of interest was manually defined to encompass the entire cross-sectional area of the nerve. Using the device’s automatic elastography software, tissue stiffness within the selected region of interest was quantitatively recorded in kilopascals (Fig. 2). These measurements were considered objective parameters reflecting the elastic properties of the median nerve. To enhance measurement reproducibility, three measurements were obtained from each hand, and the arithmetic mean of these values was used for statistical analysis. Shear wave elastography measurements were similarly obtained at the carpal tunnel inlet to ensure consistency across imaging modalities and to minimize variability related to anatomical heterogeneity along the tunnel. Measurements were intentionally limited to a single standardized anatomical level to improve reproducibility and comparability between participants, as multi-level assessment may increase measurement variability, particularly in paediatric patients. Formal interobserver reliability analysis was not performed due to the limited sample size and the use of consensus-based final measurements; however, all examinations were conducted by radiologists with substantial experience in musculoskeletal ultrasonography and elastography.

### Statistical analysis

Statistical analyses were performed using R software (version 4.1.2). Normality assumptions were assessed using the Shapiro–Wilk test and *Q*–*Q* plots, while homogeneity of variances was evaluated with Levene’s test. Numerical variables were presented as mean±standard deviation for normally distributed data and as median with interquartile range (first–third quartiles) for non-normally distributed data. Categorical variables were reported as frequencies and percentages. For comparisons among the three groups (control, carpal tunnel syndrome-negative mucopolysaccharidosis, and carpal tunnel syndrome-positive mucopolysaccharidosis), Welch’s ANOVA was used for approximately normally distributed variables, and Tamhane’s T2 test was applied for post hoc multiple comparisons. For non-normally distributed variables, the Kruskal–Wallis *H* test was performed, followed by Bonferroni-adjusted Dunn’s test for multiple pairwise comparisons of statistically significant results. The diagnostic accuracy of median nerve cross-sectional area, vascular index and shear wave elastography-derived stiffness values (kilopascal) was evaluated using receiver operating characteristic (ROC) curve analysis. The area under the ROC curve (AUC) was reported with 95% confidence intervals. Optimal cut-off values were determined using the Youden index. For each parameter, sensitivity, specificity, positive predictive value, and negative predictive value were calculated with 95% confidence intervals. A *P*-value <0.05 was considered statistically significant in all analyses.

## Results

A total of 44 participants aged between 3 years and 18 years (mean age 11.6±4.8 years) were included in the study, comprising 22 patients with mucopolysaccharidosis and 22 age- and sex-matched healthy controls (Table 1). Within the mucopolysaccharidosis group, six patients had electromyography-confirmed bilateral carpal tunnel syndrome, while the remaining patients had no electrophysiological evidence of carpal tunnel syndrome.

**Table 1:** Sociodemographic and clinical characteristics of the patients

Characteristics	Value
Age (years)	11.59±4.75
Sex (boy/girls)	22 (50%)/22 (50%)
Control group	22 (50%)
MPS+CTS - negative	16 (36.4%)
MPS- CTS - positive	6 (13.6%)
MPS group	22 (50%)
MPS - type I	1 (2.3%)
MPS - type I-HS	2 (4.5%)
MPS - type II	2 (4.5%)
MPS - type IIIA	1 (2.3%)
MPS - type IIIC	1 (2.3%)
MPS - type IVA	9 (20.4%)
MPS - type VI	5 (11.4%)
MPS - type VII	1 (2.3%)

Data are presented as mean±standard deviation or number (percentage, %)

*CTS* carpal tunnel syndrome, *MPS* mucopolysaccharidosis

In the comparative analyses among the control group, carpal tunnel syndrome-negative mucopolysaccharidosis patients, and carpal tunnel syndrome-positive mucopolysaccharidosis patients, statistically significant differences were observed in median nerve cross-sectional area (CSA), VI, and elastography stiffness (kilopascal, kPa) values (*P*<0.001). All parameters increased progressively from controls to carpal tunnel syndrome-negative and carpal tunnel syndrome-positive mucopolysaccharidosis groups. For example, the mean right median nerve CSA was 5.36 mm^2^ in the control group, 6.60 mm^2^ in the carpal tunnel syndrome-negative mucopolysaccharidosis group, and 9.50 mm^2^ in the carpal tunnel syndrome-positive mucopolysaccharidosis group (Table 2).

**Table 2:** Comparison of clinical parameters among study groups

Parameters	Control (*n*=22)	MPS/CTS-negative (*n*=16)	MPS/CTS-positive (*n*=6)	*P*-value
Right nerve CSA (mm^2^)	5.36±0.69ᵃ	6.60±1.31ᵇ	9.50±1.94ᶜ	<0.001^d^
Left nerve CSA (mm^2^)	5.36±0.70ᵃ	6.55±1.16ᵇ	9.65±2.00ᶜ	<0.001^d^
Right vascular index	1.80 [1.60–2.00]ᵃ	3.65 [3.18–4.63]ᵇ	10.05 [8.05–11.53]ᵇ	<0.001^e^
Left vascular index	1.80 [1.63–2.10]ᵃ	3.75 [3.22–4.63]ᵇ	10.15 [7.42–11.75]ᵇ	<0.001^e^
Right SWE (kPa)	11.32±2.12ᵃ	20.88±3.20ᵇ	40.83±8.77ᶜ	<0.001^d^
Left SWE (kPa)	10.96±2.21ᵃ	21.02±2.91ᵇ	44.00±10.58ᶜ	<0.001^d^

Values are presented as mean±standard deviation or median [interquartile range]. Different superscript letters within the same row indicate statistically significant differences

*CTS* carpal tunnel syndrome, *MPS* mucopolysaccharidosis, *SWE* shear wave elastography

^d^Welch’s *F* test

^e^Kruskal–Wallis *H* test

**Fig. 3:**
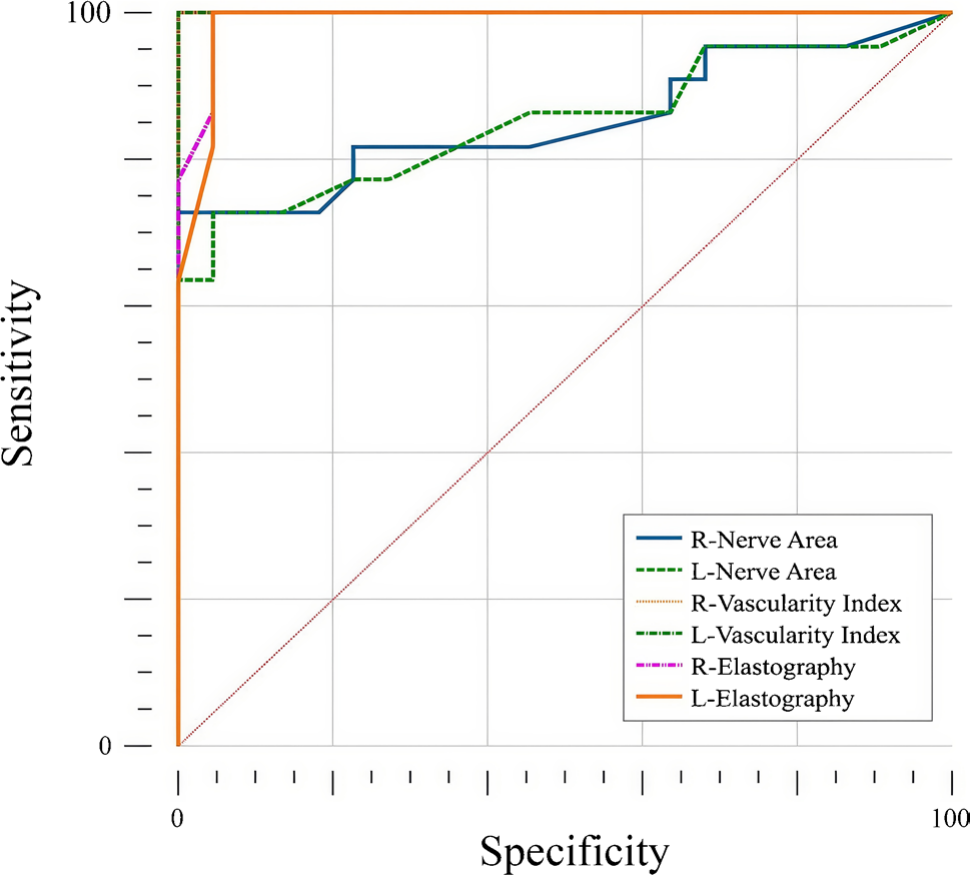
Receiver operating characteristic curves demonstrating the diagnostic performance of median nerve cross-sectional area, vascular index, and shear wave elastography–derived stiffness values for discriminating patients with mucopolysaccharidosis from healthy controls

The diagnostic accuracy of median nerve CSA, VI, and kPa in distinguishing patients with mucopolysaccharidosis from healthy individuals was evaluated using ROC curve analysis. Accordingly, the area under the curve (AUC) values for right and left nerve CSA were 0.853 and 0.851, respectively; the vascular index yielded an AUC of 1.000 for both sides; and elastography demonstrated AUC values of 0.992 on the right and 0.988 on the left. All parameters showed statistically significant diagnostic performance with high sensitivity and specificity (all *P*-values <0.001) (Table 3, Fig. 3).

**Table 3:** Diagnostic performance of nerve CSA, vascular index, and SWE parameters for differentiating mucopolysaccharidosis patients from healthy controls

Parameter	CSA (right)	CSA (left)	VI (right)	VI (left)	SWE (right)	SWE (left)
AUC (95% CI)	0.853 (0.714–0.942)	0.851 (0.712–0.940)	1.000 (0.920–1.000)	1.000 (0.920–1.000)	0.992 (0.904–1.000)	0.988 (0.897–1.000)
*P*-value	<0.001	<0.001	<0.001	<0.001	<0.001	<0.001
Cut-off value	>6.3 mm^2^	>6.4 mm^2^	>2.3	>2.4	>13 kPa	>13 kPa
Sensitivity (%)	72.7 (49.8–89.3)	72.7 (49.8–89.3)	100 (84.6–100)	100 (84.6–100)	100 (84.6–100)	100 (84.6–100)
Specificity (%)	100 (84.6–100)	95.4 (77.2–99.9)	100 (84.6–100)	100 (84.6–100)	95.4 (77.2–99.9)	95.4 (77.2–99.9)
Positive predictive value (%)	100	94.1 (69.9–99.1)	100	100	95.7 (76.4–99.3)	95.7 (76.4–99.3)
Negative predictive value (%)	78.6 (65.0–87.9)	77.8 (63.7–87.4)	100	100	100	100

Values are presented with 95% confidence intervals

*AUC* area under the curve, *CI* confidence interval, *CSA* cross-sectional area, *SWE* shear wave elastography, *VI* vascular index

**Fig. 4:**
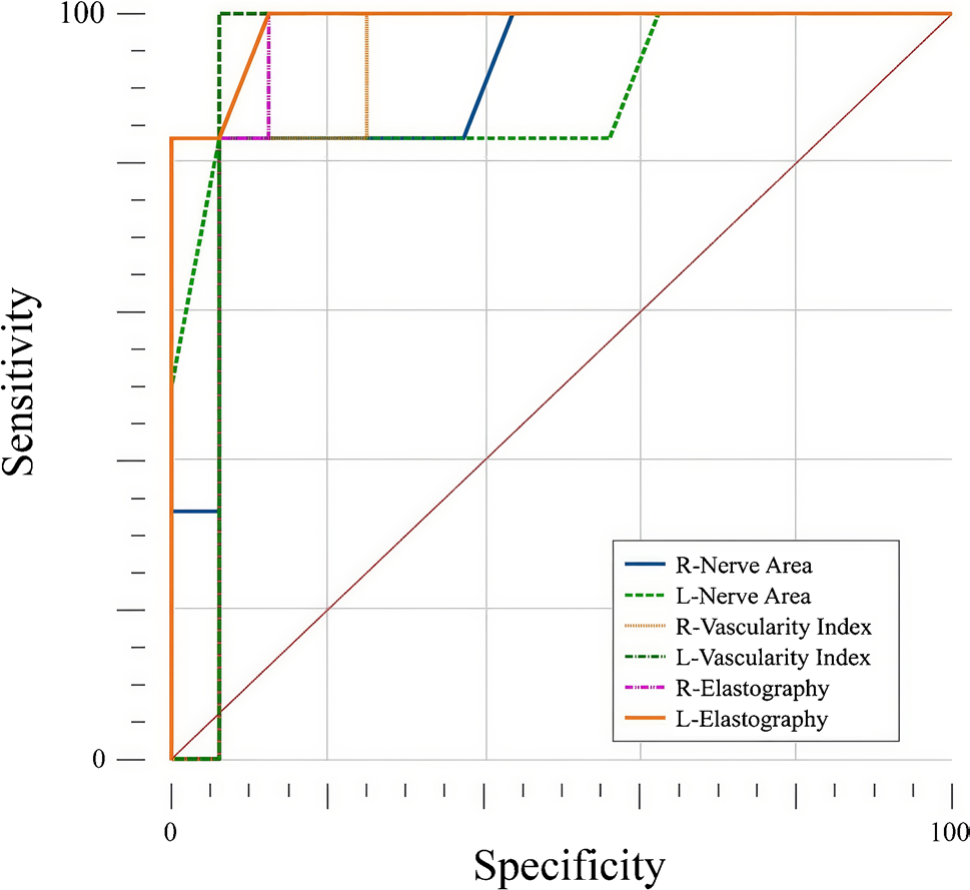
Receiver operating characteristic analyses of median nerve cross-sectional area, vascular index, and shear wave elastography–derived stiffness values for differentiating carpal tunnel syndrome–positive and carpal tunnel syndrome–negative patients with mucopolysaccharidosis

Additionally, among individuals with mucopolysaccharidosis only, the discriminatory power of the same parameters according to the presence of carpal tunnel syndrome was further examined. ROC analysis revealed significant differences between carpal tunnel syndrome-positive and carpal tunnel syndrome-negative individuals for nerve CSA (AUC 0.901 right, 0.891 left), vascular index (AUC 0.906 right, 0.938 left), and elastography (AUC 0.979 right, 0.984 left). Sensitivity ranged from 83.3% to 100%, while specificity ranged from 87.5% to 93.7% (Table 4, Fig. 4). Detailed clinical symptomatology related to carpal tunnel syndrome was not systematically recorded for all patients; therefore, the relationship between symptom status and ultra-microangiography findings could not be formally analyzed.

**Table 4:** Diagnostic performance of nerve CSA, vascular index, and SWE for differentiating CTS-positive and CTS-negative mucopolysaccharidosis patients

Parameter	CSA (right)	CSA (left)	VI (right)	VI (left)	SWE (right)	SWE (left)
AUC (95% CI)	0.901 (0.698–0.986)	0.891 (0.684–0.982)	0.906 (0.705–0.988)	0.938 (0.747–0.996)	0.979 (0.810–1.000)	0.984 (0.818–1.000)
*P*-value	<0.001	<0.001	<0.001	<0.001	<0.001	<0.001
Cut-off value	>7.5 mm^2^	>7.7 mm^2^	>5.1	>5.2	>22 kPa	>23 kPa
Sensitivity (%)	83.3 (35.9–99.6)	83.3 (35.9–99.6)	83.3 (35.9–99.6)	100 (54.1–100)	100 (54.1–100)	100 (54.1–100)
Specificity (%)	93.7 (69.8–99.8)	93.7 (69.8–99.8)	93.7 (69.8–99.8)	93.7 (69.8–99.8)	87.5 (61.7–98.4)	87.5 (61.7–98.4)
Positive predictive value (%)	83.3 (42.0–97.2)	83.3 (42.0–97.2)	83.3 (42.0–97.2)	87.5 (47.4–97.6)	75.0 (45.1–91.6)	75.0 (45.1–91.6)
Negative predictive value (%)	93.8 (71.4–98.9)	93.8 (71.4–98.9)	93.8 (71.4–98.9)	100	100	100

Values are presented with 95% confidence intervals

*AUC* area under the curve, *CI* confidence interval, *CSA* cross-sectional area, *CTS* carpal tunnel syndrome, *SWE* shear wave elastography, *VI* vascular index

## Discussion

In this study, we evaluated the diagnostic utility of ultra-microangiography and shear wave elastography for the assessment of carpal tunnel syndrome in pediatric patients with mucopolysaccharidosis. Our results demonstrated that median nerve vascular index and shear wave elastography–derived stiffness values were significantly increased in patients with mucopolysaccharidosis compared with healthy controls, with further elevations observed in patients with electromyography-confirmed carpal tunnel syndrome. These findings indicate that quantitative ultrasonographic parameters may allow early and objective discrimination between carpal tunnel syndrome–positive and carpal tunnel syndrome–negative patients. In pediatric patients with mucopolysaccharidosis, carpal tunnel syndrome is frequently underdiagnosed due to subtle or absent clinical symptoms and the limited reliability of physical examination [13–16, 28–30]. Adult-based clinical assessment criteria and pathophysiological models are often insufficient in this population, underscoring the need for objective and quantitative imaging approaches for early diagnosis [15, 17, 29].

Cognitive impairments affecting neurodevelopment further limit reliable symptom reporting in pediatric patients, increasing the need for objective assessment methods. Although electromyography findings in pediatric carpal tunnel syndrome unrelated to mucopolysaccharidosis may resemble those observed in adults, standardized electrodiagnostic criteria for carpal tunnel syndrome secondary to mucopolysaccharidosis have not yet been established [8]. Accordingly, there is a growing need for next-generation imaging techniques that are more reliable and less dependent on patient cooperation.

Ultrasonography (US) is one of the primary imaging modalities preferred in the pediatric population because of its wide availability, noninvasive nature, and lack of ionizing radiation. Several studies have investigated the use of various ultrasonographic parameters for the evaluation of carpal tunnel syndrome in children diagnosed with mucopolysaccharidosis [31]. Bäumer et al. reported that ultrasonography has diagnostic value in the detection of carpal tunnel syndrome in individuals with mucopolysaccharidosis, while Bocsa et al. described specific ultrasonographic findings, including enlargement of the median nerve proximal to the flexor retinaculum, changes in nerve thickness at the tunnel outlet, decreased echogenicity, and increased Doppler signals [18, 28]. Koç Yekedüz et al. reported that, based on measurements using the wrist–forearm ratio, values of 1.35 or higher constituted a significant threshold for the diagnosis of carpal tunnel syndrome [31]. In addition, Abrishamchi et al. demonstrated that CSA and wrist–forearm ratio values were significantly correlated with the severity of neuropathy as determined by electromyography [22]. However, most of the parameters used in existing studies are primarily aimed at evaluating advanced stages of carpal tunnel syndrome, and their sensitivity for the preclinical or early diagnosis of the disease remains limited. In contrast, the ultrasonography-based quantitative indicators used in our study—namely the VI and shear wave elastography–derived stiffness values (kPa)—have not been addressed in previous studies; in this respect, our study aims to fill an important gap in the current literature.

To our knowledge, few studies have explored the combined use of ultra-microangiography and shear wave elastography for the evaluation of carpal tunnel syndrome in pediatric patients with mucopolysaccharidosis. The VI obtained by UMA and the stiffness values expressed in kPa derived from SWE demonstrated strong potential as quantitative, observer-independent biomarkers for early diagnosis. In addition, the three-group design of the study—comprising healthy controls, carpal tunnel syndrome-negative mucopolysaccharidosis patients, and carpal tunnel syndrome-positive mucopolysaccharidosis patients—enabled a clearer delineation of diagnostic differences and disease progression. The elevated VI values observed in mucopolysaccharidosis patients with carpal tunnel syndrome were associated with increased perineural microvascular inflammation, highlighting the sensitivity of UMA in detecting vascular responses. Concurrently, the increased kPa values obtained with SWE reflected fibrotic changes secondary to glycosaminoglycan accumulation within the median nerve, allowing quantitative assessment of structural tissue stiffness.

Previous studies have shown that ultra-microangiography and shear wave elastography can provide reproducible and objective measures of microvascularity and tissue stiffness in peripheral nerves and musculoskeletal tissues [32–36]. One of the most remarkable and clinically meaningful findings of the present study is that patients with mucopolysaccharidosis who had not yet developed clinically overt carpal tunnel syndrome could be statistically distinguished from healthy individuals. This finding indicates that advanced ultrasonographic techniques such as UMA and SWE are capable of detecting subclinical microvascular and fibrotic changes within the median nerve with high sensitivity, even before the onset of carpal tunnel syndrome-specific symptoms. In particular, the significant increases observed in vascular index and tissue elasticity measurements allow noninvasive monitoring of early inflammatory processes and fibrotic remodeling triggered by glycosaminoglycan accumulation in nerve tissue associated with mucopolysaccharidosis. Moreover, the strong concordance between UMA and SWE parameters and electromyography findings enabled a precise diagnostic differentiation between carpal tunnel syndrome-positive and carpal tunnel syndrome-negative mucopolysaccharidosis subgroups. These findings suggest that quantitative ultrasonographic parameters may reflect the severity of neuropathic involvement along a continuum of disease progression. It should be acknowledged that enzyme replacement therapy does not modify disease manifestations to the same extent across all mucopolysaccharidosis subtypes, particularly with respect to musculoskeletal and peripheral nerve involvement. In the present study, detailed data regarding treatment duration and subtype-specific treatment effects were not uniformly available; therefore, the potential influence of treatment-related factors on the imaging findings could not be systematically assessed.

This study has several limitations. First, owing to the rare nature of mucopolysaccharidosis, the overall sample size and the number of patients within individual subtypes were limited, which restricts the performance of subgroup analyses and the generalizability of the findings. The single-center design of the study similarly represents a limitation to its external validity. Although nerve conduction studies are known to occasionally yield false results, electromyography was not performed in the control group, and the absence of carpal tunnel syndrome in these individuals was assumed based solely on physical examination findings. Clinical symptoms related to carpal tunnel syndrome were not systematically recorded for all patients; therefore, correlations between symptom status and ultra-microangiography findings could not be evaluated. In addition, although electromyography was used as the reference standard for the diagnosis of carpal tunnel syndrome, electrodiagnostic testing may yield false-negative results, particularly in early-stage disease, and does not provide direct information regarding the underlying pathophysiological mechanisms of neuropathy. Furthermore, intra- or interobserver reliability analysis using the intraclass correlation coefficient (ICC) could not be performed to assess the reproducibility of ultrasonographic measurements, due to both the limited number of patients and the fact that imaging was conducted by a small number of experienced operators. Although UMA and SWE provide quantitative parameters, the lack of widely established age-specific reference ranges for VI and kPa values in the pediatric population may complicate the interpretation of the findings. Furthermore, owing to the cross-sectional design of the study, temporal changes in UMA and SWE parameters, disease progression, and treatment response could not be evaluated. Despite these limitations, we believe that our study makes an important contribution to the existing literature by highlighting the potential role of quantitative ultrasonographic applications in the early diagnosis of carpal tunnel syndrome in patients with mucopolysaccharidosis.

In conclusion, the combined use of ultra-microangiography and shear wave elastography offers complementary quantitative approaches for the assessment of median nerve alterations in mucopolysaccharidosis-related carpal tunnel syndrome. Increased vascular index and stiffness values are consistent with microvascular and structural changes of the median nerve and may indicate early neuropathic involvement. In pediatric patients with subtle or nonspecific clinical symptoms, these quantitative ultrasonographic parameters may facilitate objective identification of subclinical changes before the development of overt carpal tunnel syndrome. Further prospective multicenter studies with larger cohorts are required to establish reference values and to clarify the clinical and prognostic significance of these findings.

## Data Availability

The datasets generated and/or analyzed during the current study are not publicly available due to ethical restrictions and the need to protect patient privacy, but are available from the corresponding author on reasonable request.
